# Soft Tissue Healing After Post-Extractive Alveolar Ridge Reconstruction Using a Magnesium Membrane-Based Regenerative Approach: A Preliminary Study

**DOI:** 10.3390/jfb17070328

**Published:** 2026-07-06

**Authors:** Ilham Mounssif, Claudio Mazzotti, Valentina Bentivogli, Francesco Bondi, Diego Bianchelli, Matteo Sangiorgi, Giovanni Zucchelli, Martina Stefanini

**Affiliations:** 1 Department of Biomedical and Neuromotor Sciences, University of Bologna, 40125 Bologna, Italy; claudio.mazzotti5@unibo.it (C.M.); valentin.bentivogli3@unibo.it (V.B.); matteo.sangiorgi7@unibo.it (M.S.);; 2 Department of Periodontology, Dental School of Dental Medicine, University of Bern, 3012 Bern, Switzerland

**Keywords:** alveolar ridge augmentation, membrane, wound healing, patient-reported outcome measures, oral health, quality of life, pain, postoperative, magnesium

## Abstract

Post-extractive alveolar ridge reconstruction with resorbable barrier membranes aims to preserve ridge dimensions and support future implant placement, yet evidence on soft tissue healing with magnesium-based membranes remains limited. This prospective observational pilot study evaluated mucosal healing following alveolar ridge reconstruction using a resorbable pure magnesium membrane (NOVAMag^®^) combined with a xenogeneic bone graft and a collagen dermal matrix overlay in five consecutively enrolled patients. Soft tissue healing was assessed with the Wound Healing Index (WHI) at 7, 14, 30, 90, and 180 days postoperatively. Secondary outcomes included postoperative pain (VAS) and oral health-related quality of life (OHIP-14) at 7 days. Mean WHI scores progressed from 4.4 ± 0.8 at day 7 to 4.8 ± 0.4 at day 14, with complete excellent healing (WHI 5.0 ± 0.0) achieved in all patients by day 90 and maintained through day 180. One case of partial wound dehiscence resolved spontaneously without infection or graft loss. Mean VAS was 1.86 ± 1.89 cm, and mean OHIP-14 was 20.8 ± 5.23, indicating limited and transient patient-reported impact. These preliminary findings support the clinical feasibility of the NOVAMag^®^ membrane as part of a combined regenerative approach in post-extractive ridge reconstruction and warrant validation in larger controlled trials.

## 1. Introduction

Tooth extraction initiates a complex cascade of biological events that ultimately leads to dimensional remodeling of the alveolar ridge. Both experimental and clinical studies have regularly demonstrated that this remodeling process results in significant reductions in ridge width and height, especially affecting the buccal bone plate [1,2]. In humans, it has been estimated that nearly 50% of the ridge width reduction may occur within the first year following tooth extraction, with the majority of these dimensional changes occurring during the first three months [2]. These alterations may compromise prosthetically driven implant placement and negatively affect both functional and esthetic outcomes [3,4].

To reduce these changes, several surgical strategies have been proposed, including alveolar ridge preservation (ARP) and alveolar ridge reconstruction (ARR). These procedures aim to stabilize the post-extraction socket and maintain or restore ridge dimensions by using grafting materials combined with barrier membranes, according to the principles of guided bone regeneration (GBR) [5,6]. Barrier membranes play a key role during this process by excluding rapidly proliferating epithelial and connective tissue cells from the defect area, while allowing osteogenic cells to repopulate the regenerative compartment.

Although GBR procedures have traditionally been evaluated primarily in terms of bone regeneration outcomes, the role of soft tissue healing in determining the success of regenerative procedures is increasingly recognized. The integrity of the mucosal barrier covering the grafted site is essential for protecting the regenerative compartment from bacterial contamination and mechanical disruption. Early wound healing complications—particularly flap dehiscence and membrane exposure—remain among the most frequently reported adverse events in GBR procedures and may compromise the stability of the graft and the predictability of the regenerative outcome [7].

The biological behavior of the barrier membrane critically regulates both regenerative outcomes and soft tissue healing. Non-resorbable membranes (ePTFE, titanium-reinforced) offer excellent mechanical stability but require a second surgical procedure for removal and carry a higher risk of exposure, whereas resorbable collagen membranes provide favorable biocompatibility but may lack sufficient rigidity in large or non-contained defects [5]. In recent years, biodegradable metallic membranes based on magnesium alloys have appeared as a new class of biomaterials for oral regenerative procedures. Magnesium has several properties that make it attractive for clinical use, including high mechanical strength, biocompatibility, and predictable biodegradation [8,9]. The NOVAMag^®^ membrane (Botiss Biomaterials GmbH, Zossen, Germany) (Mg-membrane), manufactured from 99.95% pure magnesium, received its CE mark in 2021 and combines the mechanical stability of rigid barriers with the biological advantages of resorbability, eliminating the need for a second surgical retrieval procedure [8,9,10,11].

Preliminary clinical evidence is accumulating. Preclinical studies demonstrated complete Mg-membrane resorption by 16 weeks, with histomorphometric bone regeneration comparable to that in collagen controls [8]. Clinical reports describe successful staged GBR in complex post-extractive defects, with both immediate and delayed implant placement, without adverse reactions to the magnesium fixation system [9,10]. Notably, Tabanella et al. [11] documented four cases of wound dehiscence during magnesium membrane GBR; membrane exposure resolved spontaneously in all instances without infection or bone loss, and implant placement proceeded as planned—a behavior sharply distinct from that observed with non-resorbable membranes.

Beyond its mechanical role, the alkaline microenvironment generated by magnesium degradation products contrasts with the acidic pH produced by synthetic resorbable polymers (PLA/PGA), which is linked with foreign body reactions and local tissue irritation [12]. Additionally, the Mg^2+^ ions released during progressive corrosion have been shown to stimulate the keratinocyte migration pathway, promote fibroblast proliferation and myofibroblast differentiation, and enhance collagen synthesis, biological processes central to early soft tissue repair [13]. In vivo evidence further demonstrates that magnesium implant degradation exerts immunomodulatory and pro-angiogenic effects on peri-implant soft tissues, attenuating fibrosis compared to non-degradable titanium controls [14]. These properties may confer intrinsic advantages for peri-oral mucosal healing in regenerative applications.

Although these promising results are encouraging, most studies have focused on hard tissue outcomes, leaving a significant gap in the evidence base regarding soft tissue healing. Mucosal integrity is a fundamental determinant of regenerative success, as early wound complications can compromise graft stability and regenerative predictability.

Standardized clinical indices provide objective, reproducible tools for monitoring mucosal healing across biomaterials and surgical techniques [15]. The Wound Healing Index (WHI), introduced by Landry et al. [16] for periodontal surgical wounds, evaluates five parameters on a five-point ordinal scale (tissue color, bleeding on palpation, granulation tissue, suppuration, and incision margin epithelialization; 1 = very poor, 5 = excellent). The WHI has been applied across a broad range of oral regenerative procedures, including post-extractive socket preservation [17,18] and GBR with titanium mesh [19]. The combined use of WHI with patient-reported measures (VAS, OHIP-14) captures complementary dimensions of the postoperative experience [20].

Despite the promising properties of magnesium-based biomaterials, evidence regarding their influence on peri-oral soft tissue healing remains limited. In particular, clinical studies evaluating mucosal healing through standardized indices following alveolar ridge reconstruction with magnesium membranes are still scarce.

Therefore, the primary aim of the present prospective observational pilot study was to evaluate the soft tissue response following alveolar ridge reconstruction using a resorbable magnesium membrane, with particular emphasis on mucosal healing assessed through the Wound Healing Index during the postoperative healing period.

## 2. Materials and Methods

### 2.1. Study Design

This study was designed as a prospective, pilot clinical investigation to evaluate the soft-tissue healing response following alveolar ridge reconstruction with a resorbable magnesium membrane combined with bone xenograft and collagen dermal matrix. The study was conducted at the Periodontal Unit of the Department of Biomedical and Neuromotor Sciences, University of Bologna. Ethical approval was obtained from the institutional ethics committee (CE AVEC235-2024-DISP-AUSLBO), and all participants provided written informed consent before enrollment. The trial was registered on clinicaltrials.gov (NCT07646795).

Patients requiring extraction and subsequent implant-supported rehabilitation were enrolled in this pilot study.

### 2.2. Patient Selection

Patients were included if they:

- were ≥18 years of age
- required extraction of a tooth between the central incisor and second premolar
- presented with compromised post-extraction sockets [21] requiring ARR
- were candidates for delayed implant placement.

Exclusion criteria included:

- poor oral hygiene (FMPS/FMBS > 20%)
- systemic conditions affecting healing
- pregnancy or breastfeeding
- allergy to xenogeneic graft materials
- heavy smoking habits (≥10 cigarettes)

### 2.3. Surgical Procedure

**Figure 1:**
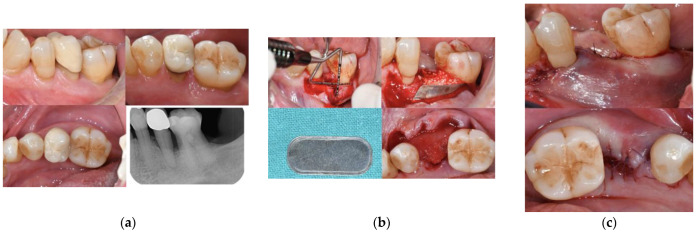
(**a**) Preoperative buccal, occlusal, lingual clinical views and x-ray showing a compromised left mandibular second premolar. (**b**) Surgical procedure involving atraumatic extraction of the tooth, Mg-membrane positioning at the buccal site, and bone grafting of the socket. Occlusal seal using CDM stabilized with PGA 7-0 sutures. (**c**) Occlusal and buccal view of flap stabilization through PGA 6-0, aiming to reach primary wound closure.

All surgical procedures (Figure 1) were performed by the same experienced operator (IM).

Following local anesthesia (articaine 4% with adrenaline 1:100,000), atraumatic tooth extraction was performed using periotomes and minimally invasive instruments to preserve the buccal plate. The socket was thoroughly debrided with curettes, and the alveolus irrigated with sterile saline. A full-thickness mucoperiosteal flap was elevated with mesial and distal releasing incisions to allow tension-free visualization of the defect and subsequent placement of regenerative materials.

The reconstruction protocol consisted of a combination of:

- xenogeneic bone graft (cerabone^®^ plus, Botiss biomaterials GmbH, Berlin, Germany)
- resorbable magnesium membrane (NOVAMag^®^ Botiss biomaterials GmbH, Berlin, Germany)
- collagen dermal matrix (mucoderm^®^ Botiss biomaterials GmbH, Berlin, Germany) (CDM)

The Mg-membrane (10 × 20 mm, 140 μm thickness; botiss biomaterials GmbH, Berlin, Germany,) was cut and shaped intraoperatively using dedicated scissors; edges were rounded with a sculptor to prevent soft tissue perforation. The membrane was positioned to overlap the defect margins by at least 3–4 mm. The CDM was adapted over the magnesium membrane to improve soft tissue adaptation and provide an additional biologic seal at the wound margin, as described by Frosecchi [10] and Blaskovic et al. [9], with 7-0 PGA suture.

The flap was stabilized in a coronal position, trying to reach primary intention closure, whenever possible, using monofilament 6-0 PGA sutures (Kalos, Nike srl, Orbetello, Italy).

Patients were instructed to take antibiotic prophylaxis, which consisted of a single preoperative loading dose of amoxicillin 2 g orally one hour before surgery, followed by amoxicillin 875 mg + clavulanate 125 mg twice daily for five postoperative days [22]. The preoperative bolus dose is supported by systematic review evidence showing that 2 g of amoxicillin administered one hour before bone augmentation procedures significantly reduces bacterial contamination of grafted particles and early failure rates [23] and reflects the protocol adopted in comparable GBR studies with the NOVAMag^®^ membrane [9,11]. Clavulanate was added to extend coverage to beta-lactamase-producing oral anaerobes prevalent in complex post-extractive environments [24]. For patients with penicillin allergy, clindamycin 600 mg preoperatively, followed by 300 mg three times daily for 5 days, is the recommended alternative [25]. Postoperative antimicrobial plaque control was prescribed as chlorhexidine gluconate 0.2% mouthwash (15 mL, 30 s), three times daily, commencing 24 h after surgery and continued for two weeks until suture removal. Sutures were removed at 14 days [26], at which point patients resumed gentle mechanical oral hygiene at the surgical site with a soft-bristled toothbrush and progressively tapered the chlorhexidine rinse over the following two weeks to avoid abrupt microbiological rebound.

### 2.4. Clinical Follow-Up

Patients were evaluated at the following post-procedure time points to monitor their healing status and overall clinical outcomes: 7–14–30–90–180 days.

At each scheduled follow-up, clinical photographs were taken, and comprehensive healing assessments were carried out to document wound closure, tissue appearance, and any complications.

### 2.5. Outcome Measures

#### 2.5.1. Primary Outcome

**Figure 2:**
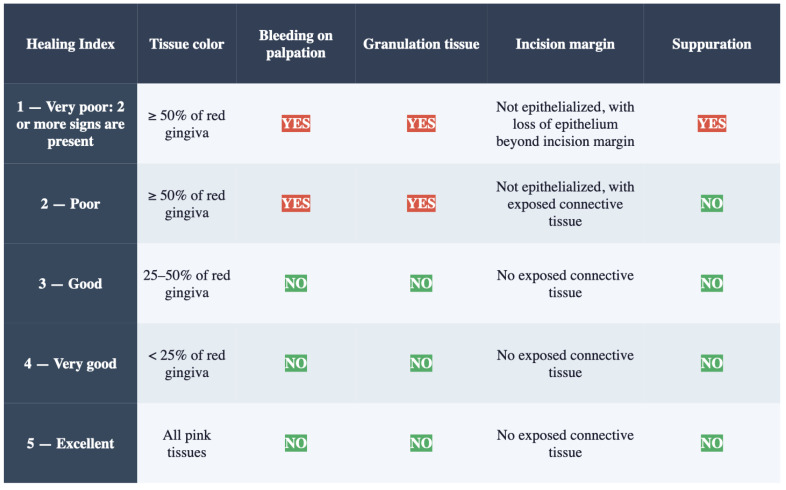
WHI [16].

Soft tissue healing was assessed using the Wound Healing Index (WHI) [16] by an expert and trained clinician (CM), who was unaware of the procedures performed (Figure 2). Prior to data collection, the examiner reviewed the WHI scoring criteria and calibrated assessments against a set of reference clinical photographs representing each score level. Formal inter-rater reproducibility assessment was not performed given the pilot nature of the study.

The WHI evaluates healing based on:

- tissue color
- presence of bleeding
- granulation tissue
- epithelialization
- suppuration.

Scores range from 1 (very poor healing) to 5 (excellent healing).

#### 2.5.2. Secondary Outcomes

Secondary outcomes included:

- Patient-reported postoperative discomfort assessed with a 10 cm visual analog scale (VAS; 0 = no pain, 100 = worst imaginable pain) recorded at 7 days postoperatively [27].
- Oral health-related quality of life assessed at 7 days using the Italian-validated version of the Oral Health Impact Profile-14 (OHIP-14) [28].

### 2.6. Statistical Analysis

Given the exploratory and pilot nature of the present study, no formal sample size calculation was performed, and no inferential statistical testing was applied. All continuous variables—including WHI scores, VAS pain scores, and OHIP-14 total scores—are expressed as means and standard deviations (mean ± SD). Categorical variables are reported as absolute frequencies and percentages. The temporal trend of WHI scores across follow-up visits (7, 14, 30, 90, and 180 days) is described narratively and graphically. Individual patient data are reported in full to ensure transparency and to allow readers to appraise the variability of outcomes within this small sample. This approach is consistent with that adopted in comparable pilot studies evaluating soft tissue healing after GBR procedures using novel biomaterials, where the primary purpose of statistical description is to generate hypotheses and estimate effect sizes to inform the design of adequately powered future randomized controlled trials [9,11]. All analyses were conducted using IBM SPSS Statistics, version 29.0 (IBM Corp., Armonk, NY, USA).

## 3. Results

This is a subset analysis of a more comprehensive protocol study approved by the local ethical committee.

Five patients were enrolled and treated according to the study protocol. Baseline demographic characteristics for all 5 patients and site locations are summarized in Table 1. Participants comprised four males and one female with a mean age of 61.0 ± 13.9 years (range 42–74). All patients were non-smokers. Treated sites included two mandibular premolars (sites 34 and 35), two mandibular first premolars (site 14, two patients), and one maxillary lateral incisor (site 12).

### 3.1. Soft Tissue Healing

**Figure 3:**
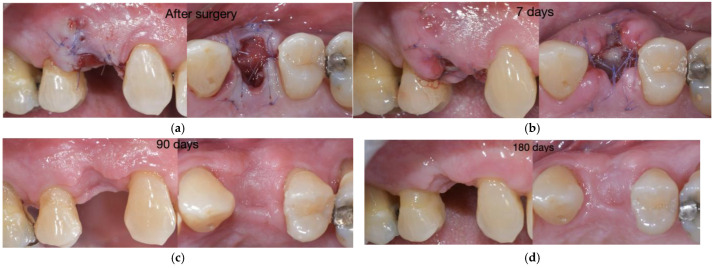
(**a**) Clinical views of the treated area of patient 5 immediately after the surgery, (**b**) at 7 days, (**c**) 90 days and (**d**) 180 days.

**Figure 4:**
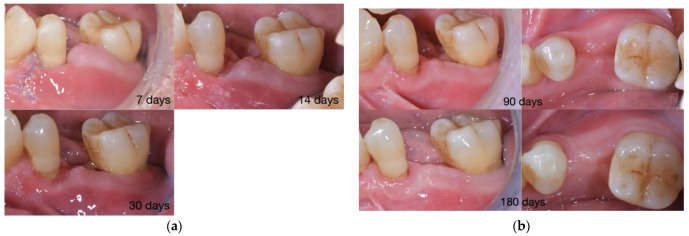
(**a**) Clinical views of the treated area at 7, 14, 30 days. (**b**) Buccal and occlusal views at 90 and 180 days.

Overall, the healing was uneventful; no adverse events related to the procedures or the biomaterial were reported by patients. WHI scores for each patient are presented in Table 2. At day 7, the mean WHI was 4.4 ± 0.8, with the mean peaking at 4.8 ± 0.4 at 14 days, with four of five patients at excellent healing. Healing was defined by minimal erythema, intact epithelial margins, and absence of exudate or granulation tissue. The single case of membrane exposure (Figure 3) yielded a lower score at the 7-day evaluation, indicating the partially open wound; subsequent follow-up visits documented progressive re-epithelialization consistent with uneven secondary healing. In keeping with the pattern recently described by Tabanella et al. [11] for wound dehiscence following magnesium membrane GBR, the exposed site showed no suppuration, no signs of local infection, and was not associated with pain. Spontaneous secondary healing with progressive fibrous tissue formation was observed at the 30-day visit. No case of graft loss, flap necrosis, or unplanned membrane removal was recorded. During subsequent follow-up visits, soft tissues appeared stable, with complete epithelialization and healthy mucosal appearance (Figure 4).

### 3.2. Patient-Reported Outcomes

Patient-reported outcomes are summarized in Table 3. Postoperative discomfort was limited and transient. VAS scores at day 7 ranged from 0 to 5 cm (mean 1.86 ± 1.89 cm). Two patients reported no pain (VAS 0 and 0.3 cm), two mild pain (VAS 1 and 3 cm), and one moderate pain (VAS 5 cm); the latter did not correspond to the patient with wound dehiscence.

OHIP-14 total scores ranged from 15 to 27 (mean 20.8 ± 5.23), showing a moderate but transient impact on oral health-related quality of life. No patient exceeded a score of 30, the threshold typically associated with significant functional impairment in surgical populations. The two patients with the highest OHIP-14 scores (27) also had the highest VAS scores, consistent with the VAS-OHIP correlation described by Dawar et al. [20].

## 4. Discussion

The present prospective pilot study evaluated soft tissue healing following post-extractive ridge reconstruction with a combined protocol including the NOVAMag^®^ resorbable magnesium membrane in five consecutively treated patients, using the WHI as the primary clinical endpoint. The series demonstrated consistently high healing scores from as early as day 7 (mean WHI 4.4 ± 0.8), rising to 4.8 ± 0.4 at day 14 and reaching 5.0 ± 0.0 from day 90 onwards. One case of partial wound dehiscence resolved spontaneously without infection, additional treatment, or compromised regenerative outcomes. Patient-reported outcomes were reassuring, mean VAS 1.86 ± 1.89 cm and OHIP-14 20.8 ± 5.23, reflecting a limited and transient postoperative impact.

The WHI trajectory in this study compares favorably with published benchmarks using equivalent or analogous indices. Wang and De Santis [19] applied the Landry Healing Index to five patients undergoing customized titanium mesh GBR with the modified “Poncho” technique, reporting mean scores of 3.0 ± 0.47 at day 3, 3.87 ± 0.76 at day 7, 4.33 ± 1.15 at day 14, and 4.69 ± 1.06 at day 30, with a 4.3% dehiscence rate. In the present series, day-7 (4.4 ± 0.8) and day-14 (4.8 ± 0.4) WHI appeared numerically higher. In the socket preservation setting, Mozzati et al. [17] reported a modified healing index averaging 5.8 with 91% closure at 21 days using an Mg–hydroxyapatite composite, and Keranmu et al. [18] documented the Landry score of 3.94 ± 0.64 at one week in an ARP trial. The WHI values in the present series appear numerically higher than both benchmarks; however, these cross-study comparisons must be interpreted with considerable caution. Differences in patient populations, surgical techniques, follow-up protocols, and outcome assessment tools preclude any conclusions regarding the superiority of the Mg-membrane-based approach over alternative regenerative protocols. This study was not designed to demonstrate comparative efficacy.

The biological basis for this favorable mucosal profile lies in the progressive degradation mechanics of the Mg-membrane. Unlike titanium meshes (dehiscence rate 15.2–23.9% [29]) and reinforced PTFE membranes (dehiscence rate up to 45% [30]), the magnesium membrane progressively degrades in the physiological environment while temporarily preserving its space-maintaining function before being absorbed [8]. The alkaline microenvironment contrasts with the acidic pH of PLA/PGA degradation, which is associated with foreign body reactions and osteolysis [12]. The single dehiscence case (Patient 5; WHI 3 at day 7) resolved without infection or bone loss and attained WHI 5 by day 90, consistent with the four spontaneously healing dehiscence cases reported by Tabanella et al. [11]. Unlike Dawar et al. [20], who described an association between wound opening and postoperative pain, there is no concordance between the highest VAS (5 cm) and the dehiscence case.

The WHI proved to be a practical and longitudinally sensitive primary endpoint. The transient score dip at 30 days in two patients (WHI = 4) reflects expected tissue remodeling dynamics, paralleling the plateau observed by Wang and De Santis [19].

An important methodological consideration is that the favorable soft tissue healing observed in the present series cannot be attributed exclusively to the magnesium membrane itself. The regenerative protocol incorporated multiple biologically active and technique-sensitive components, including coronally advanced tension-free flap management, microsurgical suturing, xenogeneic grafting material, and the adjunctive use of a CDM, all of which may have independently contributed to wound stability and mucosal maturation. More specifically, the CDM likely provided a protective biological interface that shielded wound margins from mechanical irritation and supported re-epithelialization, potentially contributing to the favorable WHI scores from day 7. The xenogeneic graft maintained socket volume and stabilized the clot, indirectly supporting wound closure, while the magnesium membrane fulfilled its space-maintaining and GBR barrier function while generating a locally favorable microenvironment through progressive corrosion. Tension-free coronal flap advancement and microsurgical suturing further represent technique-sensitive determinants of primary intention closure, independent of membrane type. The relative contribution of each component cannot be quantified within the present single-arm design, and future controlled trials comparing the protocol with and without CDM overlay will be required to isolate their individual effects on soft tissue healing. Therefore, the present findings should be interpreted as reflecting the clinical performance of a combined regenerative approach rather than the isolated effect of the Mg-membrane.

Future controlled studies comparing magnesium membranes with and without adjunctive soft tissue matrices, under standardized flap management protocols, will be necessary to clarify the specific contribution of magnesium-based biomaterials to peri-oral soft tissue healing dynamics.

The study has inherent limitations: small sample size, single-center, single operator design, absence of a control group. An exploratory statistical design stresses feasibility assessment and individual data transparency to direct future sample size calculations. The findings provide the first structured WHI-based evidence on mucosal healing with the Mg-membrane in post-extractive ridge reconstruction. Their agreement with published benchmarks in comparable clinical contexts [17,18,19] supports biological plausibility. Further RCTs with standardized CBCT bone gain measurements at re-entry are warranted.

## 5. Conclusions

Bearing in mind the limitations mentioned above, this prospective pilot study provides preliminary evidence that post-extractive alveolar ridge reconstruction with a combined regenerative protocol including the NOVAMag^®^ resorbable magnesium membrane results in favorable soft-tissue healing at 6-month follow-up. WHI scores increased steadily from the first postoperative week, achieving excellent healing in all patients by three months and remaining stable thereafter. A single episode of partial wound dehiscence resolved spontaneously without infection or compromise of the regenerative site, which aligns with previously reported benign exposure behavior for this membrane class. Patient-reported pain and quality-of-life outcomes remained favorable throughout the observation period. These findings support the clinical feasibility of the combined regenerative approach, including Mg-membrane in a post-extractive setting, and contribute to the limited evidence on mucosal healing with magnesium-based barrier devices. Larger multicenter controlled trials with standardized radiographic bone gain measurements at re-entry are required to validate these preliminary findings and to define the complete clinical profile of this membrane in implant-oriented ridge reconstruction. Future adequately powered trials should compare the NOVAMag^®^ membrane with conventional collagen membranes under identical grafting and flap management protocols. Furthermore, to minimize confounding, future study designs should consider isolating the effect of the magnesium membrane by including a study arm without adjunctive collagen dermal matrix overlay.

## Figures and Tables

**Table 1:** Demographic and descriptive characteristics at baseline.

Patient n°	Age	Gender	Extraction Site	Site Classification	Smoking
1	49	M	34	III	No
2	42	F	12	IV	No
3	73	M	35	III	No
4	74	M	14	III	No
5	67	M	14	IV	No

**Table 2:** Wound healing index at different time points postoperatively.

	WHI	WHI	WHI	WHI	WHI
Patient n°	7 days	14 days	30 days	90 days	180 days
1	5	5	5	5	5
2	5	5	5	5	5
3	4	5	4	5	5
4	5	5	4	5	5
5	3	4	5	5	5
Mean	4.4	4.8	4.6	5	5
SD	0.8	0.4	0.49	0	0

SD: standard deviation.

**Table 3:** Oral health impact profile questionnaire and Vas pain scores at 7-day follow-up.

Patient n°	OHIP-14 (14–70)	VAS (0–10 cm)
1	27	3
2	19	0
3	15	0.3
4	27	5
5	16	1
Mean	20.8	1.86
SD	5.23	1.89

## Data Availability

Data is unavailable due to privacy/ethical restrictions. The data generated are reported above.

## Abbreviations

The following abbreviations are used in this manuscript:

ARP	Alveolar Ridge Preservation
ARR	Alveolar Ridge Reconstruction
CBCT	Cone Beam Computed Tomography
CDM	Collagen Dermal Matrix
CE	Conformité Européenne
ePTFE	Expanded Polytetrafluoroethylene
EWHI	Early Wound Healing Index
FMBS	Full Mouth Bleeding Score
FMPS	Full Mouth Plaque Score
GBR	Guided Bone Regeneration
GTR	Guided Tissue Regeneration
HI	Healing Index
OHIP-14	Oral Health Impact Profile-14
MG	Magnesium
PGA	Polyglycolic Acid
PLA	Polylactic Acid
PTFE	Polytetrafluoroethylene
RCT	Randomized Controlled Trial
SD	Standard Deviation
SPSS	Statistical Package for the Social Sciences
VAS	Visual Analogue Scale
WHI	Wound Healing Index
